# MDM2 Alters Cellular Iron Homeostasis by Promoting the Degradation of Proteins Involved in Iron Storage and Iron Export

**DOI:** 10.3390/cimb48080788

**Published:** 2026-08-02

**Authors:** Yang Shi, Jin Zhang

**Affiliations:** Comparative Oncology Laboratory, Schools of Veterinary Medicine and Medicine, University of California, Davis, CA 95616, USA; ysyshi@ucdavis.edu

**Keywords:** MDM2, iron homeostasis, FTH, FTL, FPN, Protein Degradation

## Abstract

The intracellular iron levels are tightly controlled by the coordinated action of specialized proteins that regulate iron uptake, storage, and export pathways in response to iron availability. For example, Ferroportin serves as the sole mammalian iron exporter; whereas ferritin acts as the primary intracellular iron storage complex. Although both Ferroportin and ferritin are reported to be primarily degraded through lysosomal pathways, it is possible that these proteins can also be regulated through the proteasomal pathway, which may provide a more rapid mechanism to modulate intracellular iron availability. Here, we identify MDM2 as a previously unrecognized regulator of cellular iron homeostasis. We found that MDM2 is required to maintain intracellular labile iron. Mechanistically, we found that MDM2 interacts with and promotes the degradation of both Ferroportin and ferritin heavy chain. Consequently, MDM2 exerts a critical role in modulating cellular iron retention by simultaneously suppressing iron storage and iron export pathways. These findings expand the biological functions of MDM2 beyond its established role as a p53 E3 ubiquitin ligase, revealing a previously unappreciated link between MDM2 signaling and iron metabolism.

## 1. Introduction

Iron is an essential trace element that is required for the survival and normal physiological function of nearly all living organisms. The most fundamental function of iron is to facilitate electron transfer between the ferrous (Fe^2+^) and ferric (Fe^3+^) states. This redox activity enables iron to serve as a critical cofactor for many fundamental biological processes such as mitochondrial energy production, oxygen transport, DNA synthesis, and numerous enzymatic reactions [1,2]. However, due to its strong redox activity, iron is also toxic because it can react with hydrogen peroxide through the Fenton Reaction to generate hydroxyl radicals, which are highly damaging to cellular components. Indeed, dysregulation of iron balance contributes to a wide range of human diseases, including anemia, infections, metabolic diseases, neurodegenerative disorders, and cancer.

Due to its crucial biological role, iron levels are tightly controlled at both systemic and cellular levels through a coordination of iron uptake, storage, utilization, recycling, and export. The systemic iron homeostasis is mainly controlled by the hepcidin-Ferroportin axis [3,4]. Hepcidin is a peptide hormone produced by liver; whereas ferropportin is an iron exporter. When body iron levels are high, hepcidin binds to Ferroportin and subsequently, triggers the Ferroportin’s internalization and degradation in lysosomes, resulting decreased labile iron in cells. At the cellular level, iron regulatory proteins 1 and 2 (IRP1/2) maintain iron homeostasis by post-transcriptionally modulating target genes involved in iron uptake, storage, export, and conversion [5]. For example, under low iron conditions, IRP1 and IRP2 bind to iron-responsive elements (IREs) in the 5′-UTR of ferritin mRNAs (FTH1 and FTL) to repress their translation, while simultaneously binding to IREs in the 3′-UTR of transferrin receptor 1 (TfR1) mRNA to stabilize the transcript and enhance iron uptake. Through this coordinated regulation of iron storage and iron acquisition, IRP1 and IRP2 help maintain intracellular iron homeostasis. Interestingly, although IRP1/2 are known to be degraded by FBXL5 through proteasomal degradation pathway, most iron-related proteins, such as FTH, FTL and FPN are found to be degraded through lysosomal pathway. It is therefore of interest to determine whether these proteins can also be regulated through the proteasomal pathway, which may provide a more rapid mechanism to modulate intracellular iron availability.

The E3 ubiquitin ligase MDM2 (mouse double minute 2) is the principal negative regulator of the tumor suppressor p53 [6,7]. MDM2 directly binds to p53, inhibits its transcriptional activity, and targets it for proteasomal degradation, thereby maintaining low p53 levels under basal conditions. In turn, p53 transcriptionally activates MDM2 expression, forming a negative feedback loop that tightly regulates p53 abundance and function [8]. Emerging evidence suggests that MDM2 may also be linked to iron metabolism. Previous studies have shown that MDM2 expression is regulated by cellular iron availability [9] and that, under iron-deficient conditions, MDM2 mRNA is stabilized by Iron Regulatory Protein 2 (IRP2), a key post-transcriptional regulator of iron homeostasis [9,10]. However, whether MDM2 directly participates in the regulation of cellular iron metabolism remains unclear. Here, we identify MDM2 as a previously unrecognized regulator of iron homeostasis. Our data indicated that MDM2 maintains intracellular labile iron levels potentially through the proteasomal degradation of Ferroportin and ferritin heavy chain.

## 2. Materials and Methods

### 2.1. Reagents

Anti-MDM2 (Cat#86934S), anti-GAPDH (Cat# 5174S), and Anti-FTH (Cat #4393S) were purchased from Cell Signaling Technology. Anti-Ferroportin was purchased from Alpha Diagnostic, San Antonio, TX, USA (Cat# MTP11-P). Mouse anti-Flag (Cat# F1804) were purchased from MilliporeSigma, Merck KGaA, Darmstadt, Germany. Anti-Flag (Cat# 80801-2-RR), anti-FTL (Cat #10727-1-AP), and ChromoTek DYKDDDDK Fab-Trap Agrose (Cat# ffa) were purchased from Proteintech, Rosemont, IL, USA. Protein A/G Magnetic Beads (Cat# HY-k0202), proteinase inhibitor cocktail (Cat#HY-k0011), MG132 (HY-13259) and Lactacystin (HY-16594) were purchased from MedChemExpress, Monmouth Junction, NJ, USA. The WesternBright Sirius HRP substrate (Cat #K12043-D20) was purchased from Advansta, San Jose, CA, USA. JetPRIME transfection reagent (Cat #101000046) was purchased from Polyplus, Illkirch, France.

### 2.2. Cell Culture and Cell Line Generation

Mouse normal liver FL83B cells, HLF, and 293T cell line were acquired from ATCC and utilized at passages below 20. p53^−/−^ HCT116 cell line was obtained from Dr. Bert Vogelstein’s group [11,12]. Huh7 cells were obtained from JCRB Cell bank. p53-KO and p53/Mdm2-DKO MEFs were obtained from Dr. Guillermina Lozano’s group. The p53/MDM2-DKO HCT116 cell line was generated by Crispr-cas9 technology as previously described [13]. Briefly, p53^−/−^ HCT116 cells were co-transfected with two plasmids expressing guide RNA targeting MDM2, followed by puromycin selection for 3 wk. Individual clones were picked and confirmed by Western blot analysis. All the cells and their derivatives were cultured in DMEM (Dulbecco’s modified Eagle’s medium), (Life Technology, Waltham, MA, USA) supplemented with 10% fetal bovine serum. MEFs were cultured in DMEM supplemented with 10% FBS, 55 μM β-mercaptoethanol, and 1× non-essential amino acids (NEAA) solution (Cellgro, Manassas, VA, USA).

### 2.3. Plasmids

pcDNA3 plasmid expressing MDM2 or Flag-tagged MDM2 were generated previously [14]. pcDNA3 vector expressing HA-tagged Ubiquitin was generated previously [15]. pcDNA3 vector expressing Flag-tagged Ferroportin or Ferritin heavy Chain was obtained from Genscript. To generate pcDNA3 vector expressing various FTH deletion mutants, two-step PCR strategy was used. Briefly, the first step was performed to amplify two DNA fragments by using pcDNA3 vector expressing Flag-tagged FTH as a template. Fragment #1 was amplified with a common forward primer and specific mutant reverse primer. Fragment #2 was amplified with a specific mutant delete primer and common reverse primer. The second PCR was performed using Fragment 1 and 2 as template along with common Forward and Reverse primer. The resulting PCR product was then cloned into pcDNA3 vector via EcoRI and XhoI. The common Forward primer was 5′-CTG AAT TCG CCA CCA TGA CGA CCG CGT CCA CCT CGC AGG TG-3′. The common reverse primer was 5′-GAC TCG AGT TAA GCG TAG TCA GGT ACG TCG TAA GGG TAA GCG TAA TCC GGA ACG TCG TAC GGA TAC GCG TAG TCT GGA ACG TCA TAT GGG TAG CTT TCA TTA TCA CTG TCT CCC AG-3′. The specific primers for FTH(Δ12–40) were a forward primer, 5′-CCA CCT CGC AGG TGC GCC AGT ACT TTG ACC GCG ATG ATG T-3′, and a reverse primer, 5′-ACA TCA TCG CGG TCA AAG TAC TGG CGC ACC TGC GAG GTG G-3′. The specific primers for FTH(Δ41–80) were a forward primer, 5′-TTT ACC TGT CCA TGT CTT ACA TCT TCC TTC AGG ATA TCA A-3′, and a reverse primer, 5′-TTG ATA TCC TGA AGG AAG ATG TAA GAC ATG GAC AGG TAA A-3′. The specific primers for FTH(Δ81–120) were a forward primer, 5′-AGA ACC AAC GAG GTG GCC GAC TGG CCA CTG ACA AAA ATG A-3′, and a reverse primer, 5′-TCA TTT TTG TCA GTG GCC AGT CGG CCA CCT CGT TGG TTC T-3′. The specific primers for FTH(Δ121–160) were a forward primer, 5′-CAC TAC TGG AAC TGC ACA AAG CGC CCG AAT CTG GCT TGG C-3′, and a reverse primer, 5′-GCC AAG CCA GAT TCG GGC GCT TTG TGC AGT TCC AGT AGT G-3′. To generate a vector expressing a single-guide RNA (sgRNA) targeting human MDM2, two 25-nt oligos were annealed and then cloned into pSpCas9 as previously described [16]. The sequence for MDM2 gRNA#1 was 5′-GTG GTT ACA GCA CCA TCA GT-3′. The sequence for MDM2 gRNA#2 was 5′-AGC TTC GGA ACA AGA GAC CC-3′.

### 2.4. Transient Transfection

SiRNA transfection was performed with RNAiMAX according to user’s manual. The sequence for scrambled siRNA was 5′-GCA GUG UCU CCA CGU ACU AdTdT-3′. The sequence for Mdm2 siRNAs were 5′-AAG CAG UAG CAG UGA AUC dTdT-3′ and 5′-GGA AGA AAC CCA AGA CAA AdTdT-3′.

### 2.5. In Vivo Ubiquitin Assay

In vivo ubiquitination assay was performed as described [17]. The 293T cells were transfected with indicated plasmids and treated with 5 μM MG132 for 6 h prior to harvest. Cell lysates were immunoprecipitated with anti-Flag antibody followed by Western blot with Flag antibody to detect Ferroportin or FTH ubiquitination.

### 2.6. Western Blot and Immunoprecipitation (IP) Analyses

Western blot analysis was performed as previously described [18]. Briefly, whole-cell lysates were resolved on 8–12% SDS–polyacrylamide gels and then transferred to nitrocellulose membranes. Following a 20 min incubation in blocking buffer (5% non-fat dry milk in PBST), membranes were probed with primary at 4 °C overnight and secondary antibodies for 2 h at room temperature. Blots were developed using enhanced chemiluminescence (ECL) reagents, and images were visualized using VisionWorks LS software (version 8.0; Analytik Jena, Jena, Germany).

For IP analysis, Cells were lysed in an IP lysis buffer (1% NP-40, 50 mM Tris-HCl (pH 8.0), 150 mM NaCl, and 1 mM EDTA) supplemented with proteinase inhibitor cocktail. The cell lysates were incubated overnight at 4 °C with 1 µg of primary antibody and protein A/G magnetic beads. After washing the beads eight times with IP lysis buffer, the immunocomplexes were analyzed via Western blot to detect the proteins of interest.

### 2.7. Measurement of Intracellular Labile Iron

Intracellular labile iron levels were quantified using the QuantiChrom Iron Assay Kit (BioAssay Systems, Hayward, CA, USA) according to the manufacturer’s instructions. Briefly, 50 μL of cell lysates were incubated with 200 μL of working reagent in a clear-bottom 96-well microplate for 40 min at room temperature in the dark. The optical density (OD) of the stable chromogenic complex was captured spectrophotometrically at a wavelength of 590 nm utilizing a microplate reader (Bio-Rad, Hercules, CA, USA). Labile iron concentrations were calculated using a linear standard curve generated from serial dilutions of the iron standard.

## 3. Results

### 3.1. MDM2 Is Required to Maintain Intracellular Labile Iron

**Figure 1:**
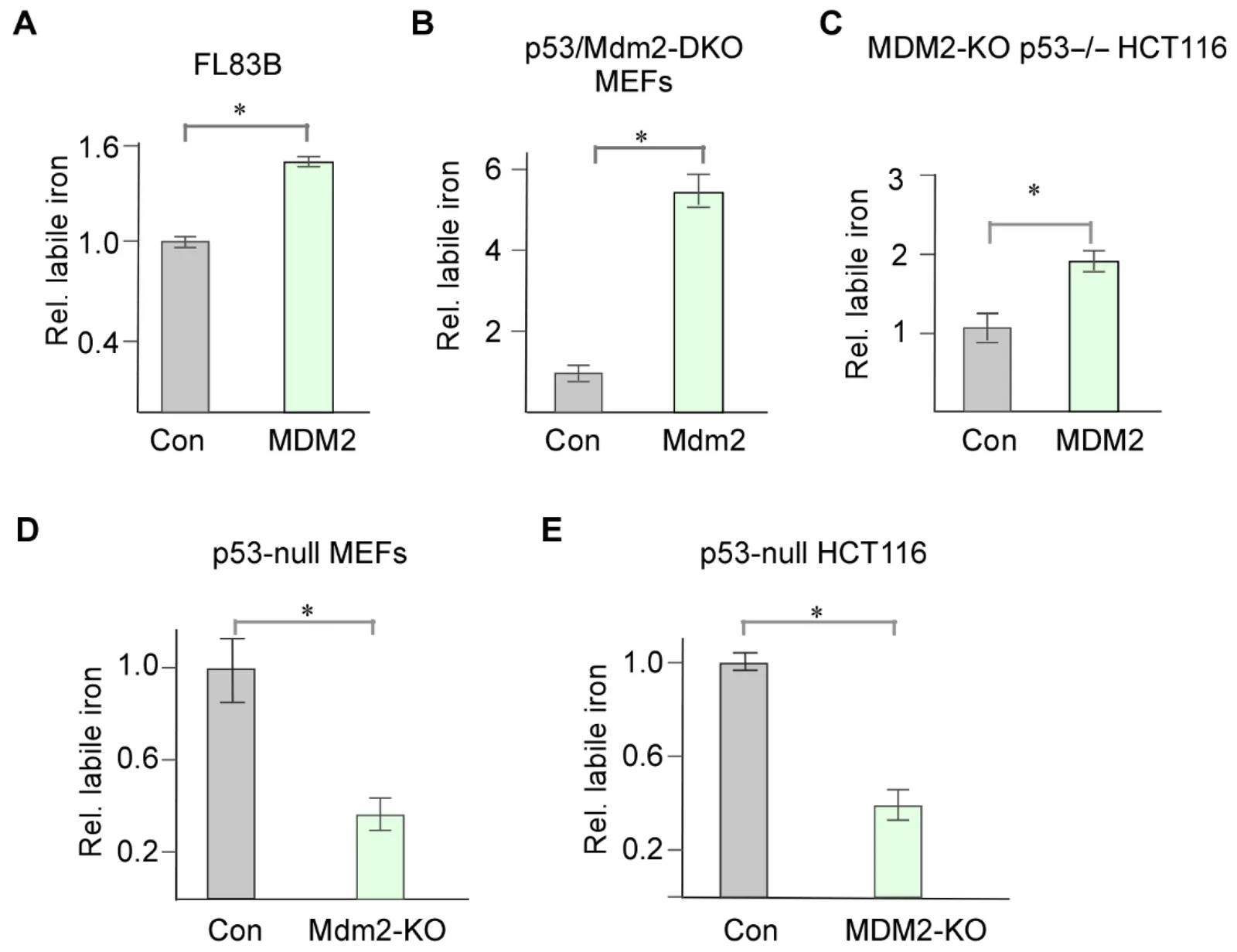
MDM2 is required to maintain intracellular labile iron. (**A**–**C**) The level of labile iron was measured in FL83B (**A**), *p53*^−/−^; *Mdm2*^−/−^ MEFs (**B**) and MDM2-KO p53^−/−^ HCT116 (**C**) cells with or without ectopic expression of MDM2. * indicated that *p* < 0.05 by Student’s *t*-test. (**D**,**E**) The level of labile iron was measured in p53^−/−^ MEFs (**D**) and p53^−/−^ HCT116 cells (**E**) with or without knockout of MDM2. * indicated that *p* < 0.05 by Student’s *t*-test.

MDM2 expression was found to be decreased by iron overload but increased by iron depletion [9,10]. However, the role of MDM2 in iron homeostasis remains to be elucidated. To determine this, FL83B, an immortalized mouse liver cell line [19], was transfected with a control plasmid or plasmid expressing MDM2, followed by QuantiChrom Iron assay to measure labile iron. We found that the level of labile iron was increased by ectopic expression of MDM2 in FL83B cells (Figure 1A). MDM2 is a negative regulator of tumor suppressor p53, which is also known to regulate cellular iron levels [20,21]. Thus, to rule out the potential effect of p53, p53/Mdm2 double-knockout (DKO) MEFs and HCT116 cells were used. We found that upon over-expression of MDM2, the level of labile iron was markedly increased in both p53/MDM2 double-knockout (DKO) MEFs and HCT116 cells (Figure 1B,C), suggesting that MDM2 modulates iron homeostasis independent of p53. To further verify the role of MDM2 in modulating iron homeostasis, the level of labile iron was measured in p53-KO and p53/MDM2-DKO MEFs. We found that when compared to p53-KO MEFs, the level of labile iron was decreased in p53/Mdm2-DKO MEFs (Figure 1D). Consistently, we also found that labile iron was decreased in p53/Mdm2-DKO HCT116 cells as compared to p53-KO HCT116 cells (Figure 1E). Together, these data indicated that MDM2 is required for maintaining labile iron in p53-indpendent manner.

### 3.2. MDM2 Targets FPN for Proteasome Degradation

**Figure 2:**
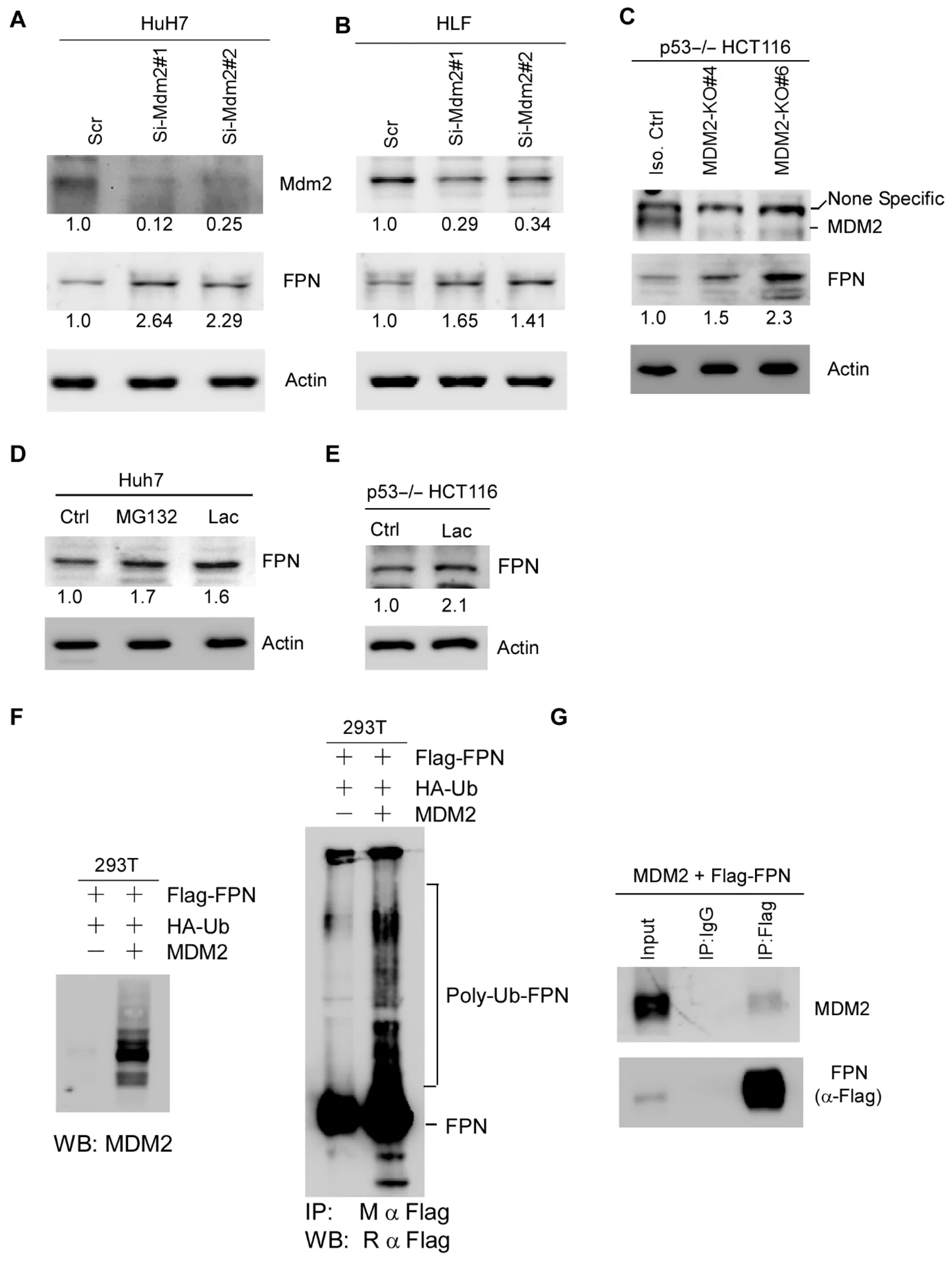
MDM2 targets FPN for proteasome degradation. (**A**,**B**) Huh7 and HLF cells were transiently transfected with scrambled and MDM2 siRNAs for 3 days, followed by Western blot analysis to detect MDM2, FPN and actin. The protein level in control was arbitrarily set as 1.0 and the relative fold change in protein was shown below each lane. (**C**) The level of FPN proteins was measured in isogenic control and MDM2-KO p53^−/−^ HCT116 cells. The protein level in control was arbitrarily set as 1.0 and the relative fold change in protein was shown below each lane. (**D**,**E**) Huh7 (**D**) and p53^−/−^ HCT116 cells were mock-treated or treated with MG132 (5 μM) or Lactacystin (5 μM) for 5 h, followed by Western blot to detect expression of FPN or Actin. The protein level in control was arbitrarily set as 1.0 and the relative fold change in protein was shown below each lane. (**F**) The 293T cells were transiently transfected with plasmids expressing Flag-tagged FPN, HA-tagged Ub, along with or without MDM2. The cell lysates were then used for Western blot analysis to detect MDM2 (**Left panel**). The cell lysates were immunoprecipitated with mouse anti Flag antibody overnight, followed by Western blot to detect Flag-FPN using rabbit anti Flag (**Right panel**). (**G**) The 293T cells were transiently transfected with plasmids expressing Flag-tagged FPN and MDM2. The cell lysates were immunoprecipitated with anti-Flag, followed by Western blot analysis to detect MDM2 and FPN.

Cellular labile iron is tightly regulated through coordinated control of iron uptake, storage, utilization, recycling, and export to maintain proper iron availability while preventing iron-mediated toxicity. Cell iron export is controlled by Ferroportin, which is encoded by the SLC40A1 gene and the only known cellular iron exporter in multicellular organisms. In this regard, we examined whether MDM2 regulates Ferroportin expression. To this end, Huh7 and HLF cells, both of which are human hepatocellular carcinoma (HCC) cell lines [22,23], were transiently transfected with a scrambled or MDM2 siRNA for 3 days, followed by Western blot to detect Ferroportin proteins. As expected, the level of MDM2 protein was efficiently decreased upon transfection of MDM2 siRNA (Figure 2A,B, MDM2 panels). Notably, we found that the level of Ferroportin proteins was markedly increased by knockdown of MDM2 (Figure 2A,B, FPN panels). To verify that MDM2 represses Ferroportin expression, isogenic control and MDM2-KO p53^−/−^ HCT116 cells were used. We found that the level of Ferroportin was increased in MDM2-KO cells when compared to isogenic control cells (Figure 2C). Since MDM2 is an E3 ligase, it is possible that MDM2 targets Ferroportin for proteasomal degradation. To this end, we first examined whether Ferroportin is subjected to proteasomal degradation. Specifically, Huh7 and p53^−/−^ HCT116 cells were mock-treated or treated with MG132 [24] or Lactacysin [25], both of which are proteasomal inhibitors, followed by Western blot to detect Ferroportin expression. We found that both MG132 and Lactacystin were able to elevate Ferroportin expression (Figure 2D,E), suggesting that Ferroportin can be degraded via proteasome. Next, in vivo ubiquitin assay was performed to determine whether MDM2 ubiquitinates Ferroportin. To this end, 293T cells were transfected with plasmids expressing HA-tagged ubiquitin, Flag-tagged Ferroportin along with or without MDM2. The cell lysates were subjected to Western blot analysis to detect MDM2 expression. Interestingly, we found that MDM2 was expressed, which was also poly-ubiquitinated when detected by MDM2 antibody (Figure 2F, left panel), consistent with previous findings that MDM2 can ubiquitinate itself [26]. Next, the lysates were immunoprecipitated with Flag antibody to bring down FPN, followed by Western blot to detect FPN. We found that Ferroportin was poly-ubiquitinated when co-expressed with MDM2 (Figure 2F, right panel). To verify this, we examined whether MDM2 interacts with Ferroportin. Briefly, 293T cells were transiently transfected with plasmids expressing Flag-tagged Ferroportin and MDM2, followed by immunoprecipitation assay. We found that when using Flag antibody to bring down Ferroportin, MDM2 was present in the Ferroportin immunocomplex (Figure 2G), indicating that MDM2 associates with Ferroportin. Together, these data suggest that MDM2 can target Ferroportin for proteasomal degradation.

### 3.3. MDM2 Suppresses Ferritin Expression

**Figure 3:**
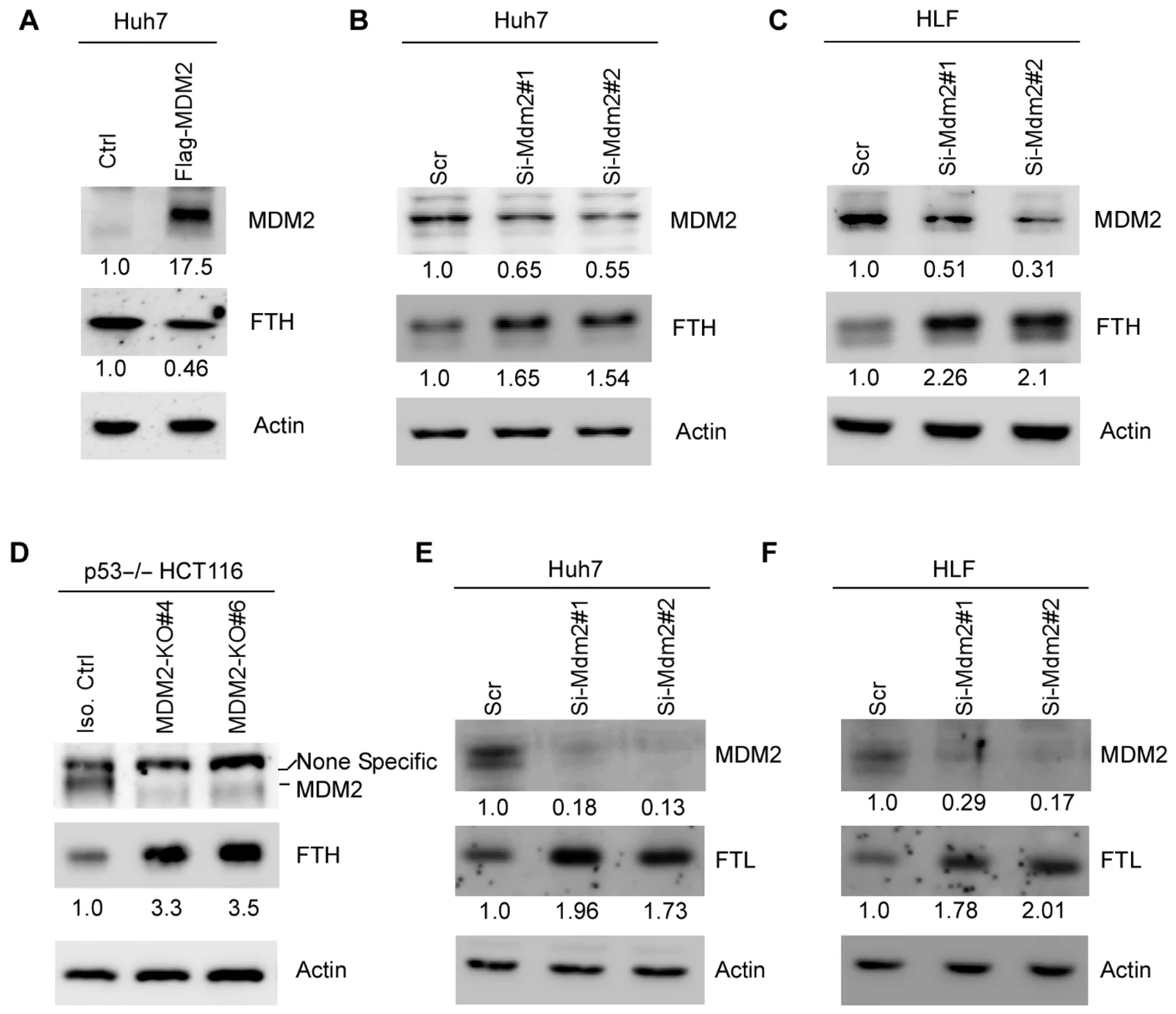
MDM2 suppresses ferritin expression. (**A**) Huh7 cells were transfected with a control plasmid or MDM2-expressing plasmid for 16 h. The cell lysates were then subjected to Western blot analysis using antibodies against MDM2, FTH and actin. The protein level in control was arbitrarily set as 1.0 and the relative fold change in protein was shown below each lane. (**B**,**C**) Huh7 and HLF cells were transiently transfected with a scrambled siRNA or siRNAs against MDM2 for 3 days. The cell lysates were used to detect MDM2, FTH, and actin by Western blot analysis. The protein level in control was arbitrarily set as 1.0 and the relative fold change in protein was shown below each lane. (**D**) Isogenic control and MDM2-KO p53^−/−^ HCT116 cells were used to detect FTH expression by Western blot analysis. The protein level in control was arbitrarily set as 1.0 and the relative fold change in protein was shown below each lane. (**E**,**F**) Huh7 and HLF cells were transfected with scrambled and MDM2 siRNA, followed by Western blot analysis to detect MDM2, FTL and Actin. The protein level in control was arbitrarily set as 1.0 and the relative fold change in protein was shown below each lane.

While Ferroportin is known to lower intracellular iron by exporting excess iron from the cell, excess iron can also be sequestered within a protein nanocage composed of ferritin heavy (FTH1) and ferritin light (FTL) chains [27,28]. Through these complementary mechanisms, the intracellular labile iron pool is tightly regulated, thereby maintaining cellular iron homeostasis. Thus, to further elucidate the role of MDM2 in iron homeostasis, we next examined whether MDM2 regulates expression of FTH and FTL. To test this, we first examine whether ectopic expression of MDM2 has an effect on FTH expression. We found that overexpression of MDM2 inhibited FTH expression in Huh7 (Figure 3A). Next, to determine whether endogenous MDM2 regulates FTH expression, two separate MDM2 siRNAs were transfected into Huh7 and HLF cells for 3 days along with a scrambled siRNA as a control. We found that the level of MDM2 protein was reduced upon siRNA transfection when compared to control siRNA (Figure 3B,C MDM2 panels). Interestingly, FTH protein levels were markedly increased upon MDM2 knockdown (Figure 3B,C, FTH panels), suggesting that MDM2 represses FTH expression. Consistent with this, we also showed that knockout of MDM2 leads to increased FTH expression in p53^−/−^ HCT116 cells (Figure 3D). Next, we examined whether MDM2 also regulates FTL expression. Consistently, FTL protein levels were increased following MDM2 knockdown (Figure 3D,E). Together, these data indicate that MDM2 suppresses the expression of key iron storage proteins, consistent with its ability to enhance the labile iron pool (Figure 1).

### 3.4. MDM2 Targets FTH for Proteasome Degradation

**Figure 4:**
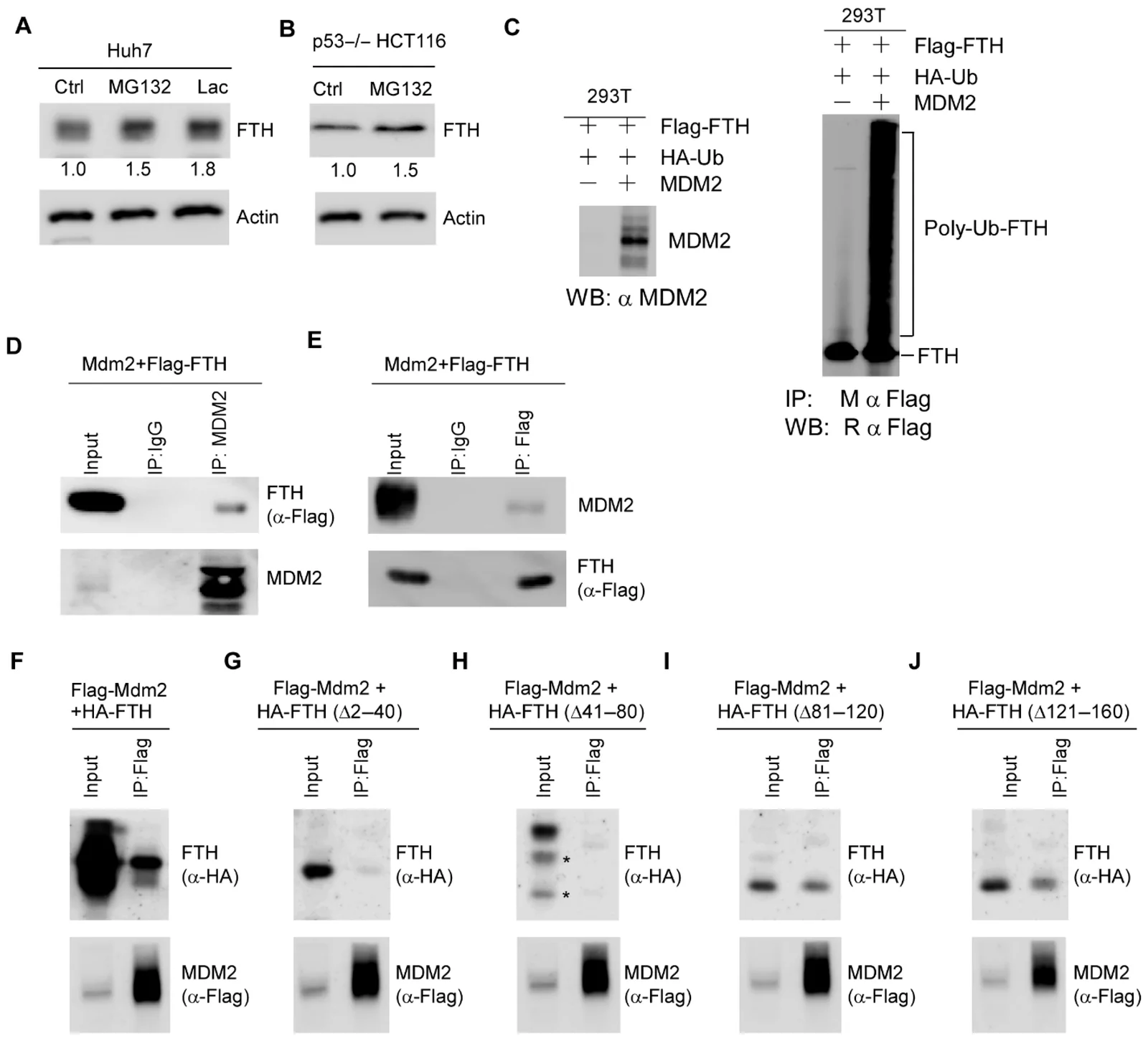
MDM2 targets FTH for proteasome degradation. (**A**) The levels of FTH and Actin proteins were measured in Huh7 mock-treated or treated with MG132 (5 μM) or Lactacystin (5 μM) for 5 h. The protein level in control was arbitrarily set as 1.0 and the relative fold change in protein was shown below each lane. (**B**) p53^−/−^ HCT116 cells were mock-treated or treated with MG132 (5 μM) for 5 h, followed by Western blot to detect expression of FTH or Actin. The protein level in control was arbitrarily set as 1.0 and the relative fold change in protein was shown below each lane. (**C**) The 293T cells were transiently transfected with plasmids expressing Flag-tagged FTH, HA-tagged Ub, along with or without MDM2. The cell lysates were then used for Western blot analysis to detect MDM2 (**Left panel**). The cell lysates were immunoprecipitated with Flag antibody overnight, followed by Western blot to detect Flag-FTH using rabbit anti Flag (**Right panel**). (**D**) The 293T cells were transiently transfected with plasmids expressing Flag-tagged FTH and MDM2. The cell lysates were immunoprecipitated with MDM2 antibody, followed by Western blot analysis to detect MDM2 and FTH. (**E**) The experiment was performed the same as in (**B**) except that Flag antibody was used for immunoprecipitation. (**F**–**J**) The 293T cells were transfected with Flag-tagged MDM2 along with HA-tagged full-length FTH (**F**), FTH(Δ2–40) (**G**), FTH (Δ41–80) (**H**), FTH (Δ81–120) (**I**) or FTH (Δ121–160) (**J**) for 16 h. The cell lysates were immunoprecipitated with Flag-Trap nanobody, followed by western with anti-Flag or anti-HA. * in (**H**) indicates non-specific bands.

FTH possesses ferroxidase activity that catalyzes the conversion of Fe^2+^ to Fe^3+^ and thus plays a direct role in regulating the intracellular labile iron pool. In contrast, FTL lacks ferroxidase activity and primarily contributes to iron nucleation and long-term iron storage within the ferritin complex. We therefore prioritized to examine whether MDM2 targets ferritin heavy chain (FTH) for degradation. To this end, we first examined whether FTH is subjected to proteasomal degradation by treating Huh7 or p53^−/−^ HCT116 cells with or without proteasomal inhibitors. We found that FTH protein levels were enhanced by MG132 or Lactacystin in both Huh7 and p53^−/−^ HCT116 cells (Figure 4A,B), indicating that FTH can be degraded by proteasome. Next, an in vivo ubiquitination assay was performed in 293T cells transfected with plasmids encoding HA-tagged ubiquitin and Flag-tagged FTH, in the presence or absence of an MDM2 expression plasmid. Cell lysates were either analyzed by Western blot to confirm MDM2 expression or subjected to immunoprecipitation with an mouse anti-Flag antibody, followed by Western blotting to detect ubiquitinated FTH using rabbit anti-Flag antibody. As expected, MDM2 was readily detected in cells co-transfected with a MDM2-expressing plasmid (Figure 4C, left MDM2 panel). Importantly, FTH showed markedly increased polyubiquitination in the presence of MDM2 (Figure 4C, right FTH panel), indicating that MDM2 promotes FTH ubiquitination. To further verify this, reciprocal IP-Western blot analysis was performed to determine whether MDM2 interacts with FTH. Briefly, 293T cells were co-transfected with plasmids expressing MDM2 and Flag-tagged FTH, followed by immunoprecipitated with Flag or MDM2 antibody. FTH was detectable in the MDM2 immunocomplex when MDM2 antibody was used for immunoprecipitation (Figure 4D). Conversely, when Flag antibody was used to bring down FTH, MDM2 was present in FTH immunocomplex (Figure 4E). To further delineate the region in FTH that interacts with MDM2, multiple HA-tagged FTH deletion mutants were generated. Next, 293T cells were co-transfected with plasmids expressing Flag-tagged MDM2 and HA-tagged Full-length FTH or deletion mutants. Cell lysates were subjected to immunoprecipitation using anti-Flag antibody, followed by Western blot analysis to detect MDM2 and FTH or its deletion mutants. We found that full-length FTH, as well as deletion mutants FTH (Δ81–120) and FTH (Δ120–160), retained the ability to interact with Flag-tagged MDM2 (Figure 4F,I,J). In contrast, FTH (Δ2–40) showed markedly reduced interaction with MDM2 (Figure 4G); whereas, FTH (Δ41–80) failed to interact with MDM2 (Figure 4H). These data indicate that the region at aa 41–80 in FTH is required to interact with MDM2.

## 4. Discussion

Iron homeostasis is maintained through coordinated regulation of iron uptake, storage, utilization, recycling, and export. In this study, we identify MDM2 as a previously unrecognized regulator of cellular iron homeostasis. Our data indicated that MDM2 promotes the degradation of both FPN and FTH, suggesting that MDM2 regulates iron metabolism through multiple mechanisms. We would like to note that degradation of FPN would suppress iron efflux and promote intracellular iron retention. Similarly, degradation of FTH would be expected to decrease iron storage capacity and thereby release labile iron. Although these effects influence distinct aspects of iron metabolism, both regulations are expected to increase the intracellular labile iron pool, consistent with our finding that MDM2 is required to maintain labile iron (Figure 1). Together, these findings suggest that MDM2 functions as a central regulator of cellular iron availability by simultaneously controlling iron storage and iron export.

Ferroportin (FPN) is the sole known cellular iron exporter in mammals and plays a central role in maintaining both systemic and cellular iron homeostasis. FPN is primarily regulated by the peptide hormone hepcidin, which binds to FPN and triggers its internalization and subsequent lysosomal degradation [29,30]. Later, it was found that ubiquitin-dependent mechanisms have also emerged as important regulators of FPN stability [31]. Indeed, RNF217 E3 ubiquitin ligase has been shown to collaborate with Hepcidin to promote FPN turnover by assembling both K48- and K63-linked polyubiquitin chains on FPN, thereby targeting the exporter for degradation through lysosomal and proteasomal pathways [32]. In the present study, we found that MDM2 also promotes FPN polyubiquitination and degradation (Figure 2). It is possible that MDM2 may also collaborate with hepcidin and thereby promotes FPN-degradation, which merits further investigation. Nevertheless, MDM2 may represent an additional layer of regulation that coordinates iron export through its effects on FPN. These findings raise the possibility that distinct E3 ligases cooperate to fine-tune FPN stability in response to specific cellular and physiological cues. Further studies are needed to determine whether MDM2 acts alone or in concert with RNF217 or hepcidin to regulate FPN turnover as well as the biological significance of MDM2-mediated FPN expression.

As the primary intracellular iron storage protein, FTH can be degraded through a selective autophagic process known as ferritinophagy, in which the cargo receptor NCOA4 delivers ferritin to lysosomes for degradation, thereby releasing stored iron into the labile iron pool [33,34,35]. In this study, we found that MDM2 represses FTH expression and polyubiquitinates FTH (Figure 4). We also found that the region at aa 41–80 in FTH is required to interact with MDM2 (Figure 4F). These data suggest that MDM2 targets FTH for proteasomal degradation. These findings may represent an alternative, rapid regulatory branch to control intracellular iron availability. In addition to MDM2, several E3 ligase, such as SMURF1 [36] and PELI1 [37], were also found to degrade FTH via proteasome-dependent pathways. Thus, proteasomal clearance of FTH may reveal a parallel responsive metabolic axis that functions independently of the classical autophagic machinery. However, several questions remain to be addressed. First, Arginine 23 of FTH has been implicated in NCOA4-dependent ferritinophagy and is critical for efficient NCOA4-mediated ferritin binding and degradation [33]. It would therefore be important to determine the specific residue(s) on FTH that is targeted by MDM2 for polyubiquitination. Second, it remains unclear how MDM2 fine-tunes FTH degradation under distinct physiological and pathological conditions. Third, MDM2 can mediate mono-ubiquitination or multi-monoubiquitination of specific substrates, thereby altering their intracellular trafficking and targeting them to the endosomal-lysosomal pathway instead of the proteasome. Given that FTH is also subject to lysosomal degradation, future studies are warranted to determine whether MDM2 regulates FTH degradation through both proteasomal and lysosomal pathways. Fourth, given that MDM2 inhibitors are currently under clinical development, it is of great interest to determine whether they can alter systemic iron homeostasis.

Tumor cells frequently exhibit increased iron demand to support DNA synthesis, mitochondrial metabolism, and sustained proliferation. The ability of MDM2 to regulate both FPN and FTH may therefore have important implications for cancer development and progression. By promoting the degradation of FTH and FPN, MDM2 may shift cellular iron homeostasis toward increased intracellular iron availability, thereby elevating the labile iron pool. While this iron-enriched state may support rapid proliferative and metabolic demands, it may also render tumor cells more susceptible to oxidative stress and iron-dependent cell death. As such, tumors with MDM2 overexpression may exhibit enhanced vulnerability to ferroptosis inducers (FINs), such as Erastin, RSL3, or IKE. In this context, ferroptosis inducers may selectively kill MDM2-high tumors by exploiting their altered iron homeostasis, while sparing normal tissues with lower MDM2 activity and more tightly regulated iron metabolism.

In this study, we identify MDM2 as a previously unrecognized regulator of cellular iron homeostasis. We found that MDM2 is required to maintain intracellular labile iron. Moreover, we found that MDM2 interacts with both Ferroportin and FTH and subsequently, promotes their degradation. Consequently, MDM2 is capable of regulating two fundamental aspects of iron metabolism: intracellular iron storage and iron export. Through coordinated control of FTH and FPN abundance, MDM2 modulates cellular iron availability and the size of the labile iron pool. Interestingly, we previously reported that IRP2 stabilizes MDM2 mRNA, thereby promoting MDM2 expression. Given the central role of MDM2 as an E3 ubiquitin ligase, it will be of considerable interest to determine whether MDM2, in turn, regulates IRP2 protein stability, potentially forming a feedback regulatory loop to fine-tune cellular iron homeostasis. Investigation of this possibility represents an important direction for future studies. Together, these findings expand the biological functions of MDM2 beyond its established role as an E3 ubiquitin ligase for p53 and reveal a previously unappreciated link between MDM2 signaling and iron homeostasis.

## Data Availability

The original contributions presented in this study are included in the article. Further inquiries can be directed to the corresponding author.

## References

[1] Ganz T., Nemeth E. (2015). Iron homeostasis in host defence and inflammation. Nat. Rev. Immunol..

[2] Hentze M.W., Muckenthaler M.U., Galy B., Camaschella C. (2010). Two to Tango: Regulation of Mammalian Iron Metabolism. Cell.

[3] Billesbolle C.B., Azumaya C.M., Kretsch R.C., Powers A.S., Gonen S., Schneider S., Arvedson T., Dror R.O., Cheng Y.F., Manglik A. (2020). Structure of hepcidin-bound ferroportin reveals iron homeostatic mechanisms. Nature.

[4] Ginzburg Y.Z. (2019). Hepcidin-ferroportin axis in health and disease. Vitam. Horm..

[5] Eisenstein R.S. (2000). Iron regulatory proteins and the molecular control of mammalian iron metabolism. Annu. Rev. Nutr..

[6] Momand J., Zambetti G.P., Olson D.C., George D., Levine A.J. (1992). The mdm-2 oncogene product forms a complex with the p53 protein and inhibits p53-mediated transactivation. Cell.

[7] Montes de Oca Luna R., Wagner D.S., Lozano G. (1995). Rescue of early embryonic lethality in mdm2-deficient mice by deletion of p53. Nature.

[8] Manfredi J.J. (2010). The Mdm2-p53 relationship evolves: Mdm2 swings both ways as an oncogene and a tumor suppressor. Gene Dev..

[9] Dongiovanni P., Fracanzani A.L., Cairo G., Megazzini C.P., Gatti S., Rametta R., Fargion S., Valenti L. (2010). Iron-Dependent Regulation of MDM2 Influences p53 Activity and Hepatic Carcinogenesis. Am. J. Pathol..

[10] Zhang J., Kong X.M., Zhang Y.H., Sun W.Q., Xu E.H., Chen X.B. (2020). Mdm2 is a target and mediator of IRP2 in cell growth control. Faseb J..

[11] Sur S., Pagliarini R., Bunz F., Rago C., Diaz L.A., Kinzler K.W., Vogelstein B., Papadopoulos N. (2009). A panel of isogenic human cancer cells suggests a therapeutic approach for cancers with inactivated p53. Proc. Natl. Acad. Sci. USA.

[12] Bunz F., Dutriaux A., Lengauer C., Waldman T., Zhou S., Brown J.P., Sedivy J.M., Kinzler K.W., Vogelstein B. (1998). Requirement for p53 and p21 to sustain G arrest after DNA damage. Science.

[13] Zhang J., Xu E.S., Ren C., Yang H.J., Zhang Y.H., Sun W.Q., Kong X.M.D., Zhang W.C., Chen M.Y., Huang E. (2018). Genetic Ablation of Promotes Lymphomagenesis in the Context of Mutant p53 by Downregulating PTEN. Cancer Res..

[14] Xu E., Zhang J., Chen X. (2013). MDM2 expression is repressed by the RNA-binding protein RNPC1 via mRNA stability. Oncogene.

[15] Jung Y.S., Liu G., Chen X.B. (2010). Pirh2 E3 Ubiquitin Ligase Targets DNA Polymerase Eta for 20S Proteasomal Degradation. Mol. Cell. Biol..

[16] Ran F.A., Hsu P.D., Wright J., Agarwala V., Scott D.A., Zhang F. (2013). Genome engineering using the CRISPR-Cas9 system. Nat. Protoc..

[17] Jung Y.S., Qian Y.J., Chen X.B. (2012). DNA polymerase eta is targeted by Mdm2 for polyubiquitination and proteasomal degradation in response to ultraviolet irradiation. DNA Repair..

[18] Chen X.B., Bargonetti J., Prives C. (1995). P53, through P21 (Waf1/Cip1), Induces Cyclin D1 Synthesis. Cancer Res..

[19] Breslow J.L., Sloan H.R., Ferrans V.J., Anderson J.L., Levy R.I. (1973). Characterization of the mouse liver cell line FL83B. Exp. Cell Res..

[20] Funauchi Y., Tanikawa C., Yi Lo P.H., Mori J., Daigo Y., Takano A., Miyagi Y., Okawa A., Nakamura Y., Matsuda K. (2015). Regulation of iron homeostasis by the p53-ISCU pathway. Sci. Rep..

[21] Zhang J., Chen X. (2019). p53 tumor suppressor and iron homeostasis. FEBS J..

[22] Nwosu Z.C., Battello N., Rothley M., Pioronska W., Sitek B., Ebert M.P., Hofmann U., Sleeman J., Wolfl S., Meyer C. (2018). Liver cancer cell lines distinctly mimic the metabolic gene expression pattern of the corresponding human tumours. J. Exp. Clin. Cancer Res..

[23] Nakabayashi H., Taketa K., Miyano K., Yamane T., Sato J. (1982). Growth of human hepatoma cells lines with differentiated functions in chemically defined medium. Cancer Res..

[24] Guo N., Peng Z. (2013). MG132, a proteasome inhibitor, induces apoptosis in tumor cells. Asia Pac. J. Clin. Oncol..

[25] Fenteany G., Standaert R.F., Lane W.S., Choi S., Corey E.J., Schreiber S.L. (1995). Inhibition of proteasome activities and subunit-specific amino-terminal threonine modification by lactacystin. Science.

[26] Ranaweera R.S., Yang X. (2013). Auto-ubiquitination of Mdm2 enhances its substrate ubiquitin ligase activity. J. Biol. Chem..

[27] Arosio P., Elia L., Poli M. (2017). Ferritin, cellular iron storage and regulation. IUBMB Life.

[28] Theil E.C. (2003). Ferritin: At the crossroads of iron and oxygen metabolism. J. Nutr..

[29] De Domenico I., Ward D.M., Langelier C., Vaughn M.B., Nemeth E., Sundquist W.I., Ganz T., Musci G., Kaplan J. (2007). The molecular mechanism of hepcidin-mediated ferroportin down-regulation. Mol. Biol. Cell.

[30] Nemeth E., Tuttle M.S., Powelson J., Vaughn M.B., Donovan A., Ward D.M., Ganz T., Kaplan J. (2004). Hepcidin regulates cellular iron efflux by binding to ferroportin and inducing its internalization. Science.

[31] Qiao B., Sugianto P., Fung E., del-Castillo-Rueda A., Moran-Jimenez M.J., Ganz T., Nemeth E. (2012). Hepcidin-Induced Endocytosis of Ferroportin Is Dependent on Ferroportin Ubiquitination. Cell Metab..

[32] Jiang L., Wang J.M., Wang K., Wang H., Wu Q., Yang C., Yu Y.Y., Ni P., Zhong Y.Y., Song Z.J. (2021). RNF217 regulates iron homeostasis through its E3 ubiquitin ligase activity bymodulating ferroportin degradation. Blood.

[33] Mancias J.D., Vaites L.P., Nissim S., Biancur D.E., Kim A.J., Wang X.X., Liu Y., Goessling W., Kimmelman A.C., Harper J.W. (2015). Ferritinophagy via NCOA4 is required for erythropoiesis and is regulated by iron dependent HERC2-mediated proteolysis. Elife.

[34] Hou W., Xie Y.C., Song X.X., Sun X.F., Lotze M.T., Zeh H.J., Kang R., Tang D.L. (2016). Autophagy promotes ferroptosis by degradation of ferritin. Autophagy.

[35] Gao M.H., Monian P., Pan Q.H., Zhang W., Xiang J., Jiang X.J. (2016). Ferroptosis is an autophagic cell death process. Cell Res..

[36] Xiong X., Li W., Yu C.L., Qiu M.H., Zhang Z.R., Hu C.M., Zhu S.L., Yang L., Pen H., Song X.Y. (2025). SMURF1-Induced Ubiquitination of FTH1 Disrupts Iron Homeostasis and Suppresses Myogenesis. Int. J. Mol. Sci..

[37] Zha X.M., Chen L., Tan Z.P., Wu T.C., Xiong Q.H., Wang H.H., Kuang Y.Y., Xing F., Lu A.H., Sun L.L. (2026). E3 ubiquitin ligase PELI1 promotes ferroptosis in granulosa cells in PCOS by degrading Fth1. J. Steroid Biochem..

